# Structure, evolution and expression of zebrafish cartilage oligomeric matrix protein (COMP, TSP5). CRISPR-Cas mutants show a dominant phenotype in myosepta

**DOI:** 10.3389/fendo.2022.1000662

**Published:** 2022-11-14

**Authors:** Helena Fabiana Forte-Gomez, Roberta Gioia, Francesca Tonelli, Birgit Kobbe, Peter Koch, Wilhelm Bloch, Mats Paulsson, Frank Zaucke, Antonella Forlino, Raimund Wagener

**Affiliations:** ^1^ Center for Biochemistry, University of Cologne, Cologne, Germany; ^2^ Department of Molecular Medicine, Biochemistry Unit, University of Pavia, Pavia, Italy; ^3^ Department of Pharmacology, University Clinic Cologne, Cologne, Germany; ^4^ Institute of Cardiovascular Research and Sport Medicine, German Sport University, Cologne, Germany; ^5^ Center for Biochemistry, Center for Molecular Medicine, University of Cologne, Cologne, Germany; ^6^ Dr. Rolf M. Schwiete Research Unit for Osteoarthritis, Department of Orthopedics (Friedrichsheim), University Hospital Frankfurt, Goethe University, Frankfurt, Germany

**Keywords:** zebrafish, extracellular matrix, thrombospondins, comp, chondrodysplasia

## Abstract

COMP (Cartilage Oligomeric Matrix Protein), also named thrombospondin-5, is a member of the thrombospondin family of extracellular matrix proteins. It is of clinical relevance, as in humans mutations in COMP lead to chondrodysplasias. The gene encoding zebrafish Comp is located on chromosome 11 in synteny with its mammalian orthologs. Zebrafish Comp has a domain structure identical to that of tetrapod COMP and shares 74% sequence similarity with murine COMP. Zebrafish *comp* is expressed from 5 hours post fertilization (hpf) on, while the protein is first detectable in somites of 11 hpf embryos. During development and in adults *comp* is strongly expressed in myosepta, craniofacial tendon and ligaments, around ribs and vertebra, but not in its name-giving tissue cartilage. As in mammals, zebrafish Comp forms pentamers. It is easily extracted from 5 days post fertilization (dpf) whole zebrafish. The lack of Comp expression in zebrafish cartilage implies that its cartilage function evolved recently in tetrapods. The expression in tendon and myosepta may indicate a more fundamental function, as in evolutionary distant Drosophila muscle-specific adhesion to tendon cells requires thrombospondin. A sequence encoding a calcium binding motif within the first TSP type-3 repeat of zebrafish Comp was targeted by CRISPR-Cas. The heterozygous and homozygous mutant Comp zebrafish displayed a patchy irregular Comp staining in 3 dpf myosepta, indicating a dominant phenotype. Electron microscopy revealed that the endoplasmic reticulum of myosepta fibroblasts is not affected in homozygous fish. The disorganized extracellular matrix may indicate that this mutation rather interferes with extracellular matrix assembly, similar to what is seen in a subgroup of chondrodysplasia patients. The early expression and easy detection of mutant Comp in zebrafish points to the potential of using the zebrafish model for large scale screening of small molecules that can improve secretion or function of disease-associated COMP mutants.

## Introduction

COMP is a homopentameric glycoprotein that was first isolated from different cartilage types and from the Swarm rat chondrosarcoma (1–3). At that time, it was considered to be a cartilage-specific protein. However, over the last decades a broader expression of COMP in many other mesenchymal tissues has been described, including tendon, muscle and dermis (4–6). Sequence analysis revealed that COMP belongs to the thrombospondin (TSP) family and is therefore also referred to as TSP5 (7).

COMP interacts with other extracellular matrix (ECM) components, including collagens from different collagen subfamilies (8). The binding to collagens and interconnection of ECM components makes a matrix-stabilizing function likely (9). In addition, COMP expression often correlates with fibrotic overexpression of collagens and increased matrix deposition, like in idiopathic pulmonary fibrosis and in different types of cancer (7). For fibrillar collagens, a role in collagen secretion and fibril formation has been suggested (10). In addition to these more structural functions, a binding to growth factors of the TGF-β superfamily, like TGF-β and different BMPs, has been described (11, 12). These interactions were shown to modulate the activity of the bound growth factor and their relevance has been demonstrated in chondrogenic (13) and osteogenic (14) differentiation of mesenchymal stem cells and in an animal model with aortic calcification (15). More recently, a protective role in the maintenance of the vascular ECM and the identification of COMP as a novel NOTCH ligand driving embryonic stem cell differentiation towards the smooth muscle lineage was reported (16).

COMP deficient mice do not show an obvious skeletal phenotype and it was speculated that the lack of COMP might be compensated by other members of the TSP family (17). However, the transgenic expression of mutant COMP variants in mice resulted in growth retardation and a chondrodysplasia-like phenotype (18). Mutations in COMP result in protein misfolding and accumulation in the endoplasmic reticulum, eventually leading to decreased cell proliferation and increased apoptosis (19, 20). In addition to skeletal alterations, a reduced tendon strength and a mild myopathy was reported in one of these mouse lines (21, 22).

Interestingly, the mutant mouse phenotype largely reflects the phenotype of human patients with mutations in the COMP gene. To date, more than 70 disease-causing mutations have been identified and associated with either the milder multiple epiphyseal dysplasia (MED) or the more severe pseudoachondroplasia (PSACH). The severity of the disease apparently depends on the specific mutation and it was shown in cell culture models that different mutations may cause the disease by different molecular mechanisms (23). MED and PSACH are rare diseases and currently, there is no treatment available for human patients (24).

In a first attempt to develop a treatment, transgenic mice were treated with anti-oxidant or anti-inflammatory compounds. Treatment with either resveratrol or aspirin reduced the amount of intracellularly accumulated COMP and the associated cellular stress as well as the extent of growth plate chondrocyte death (25). However, how these compounds affect the underlying disease mechanisms remains largely unclear (26).

Over the last years, zebrafish has been established as an attractive model organism to study human skeletal disorders (27). High fecundity, external development and the transparent early life stages are major advantages. In addition, the genome can be easily manipulated using CRISPR/Cas9 and the small size and fast development of the fish allows high throughput drug screening (28, 29). In zebrafish, functional analysis was so far performed only on Tsp4, the closest relative of COMP among the TSP family members (30). Interestingly, Tsp4b has been shown to be essential for ECM assembly and muscle attachment at myotendinous junctions (31). However, other members of the TSP family have not yet been investigated in detail.

In the present study, we therefore first characterized the structure of the zebrafish *comp* gene and analyzed the protein sequence and expression pattern in zebrafish. Second, we generated and phenotyped a zebrafish line in which a Comp mutation had been introduced by CRISPR/Cas9.

## Materials and methods

### Bioinformatic analysis

For the analysis of the orthologous gene loci the Ensembl *Danio rerio* (GRCz11), *Mus musculus* (GRCm39) and *Homo sapiens* (GRCh38.p13) genome databases were used. The potential signal peptide was predicted by SignalP v6.0 (https://services.healthtech.dtu.dk/service.php?SignalP-6.0) (32). Multiple sequence alignments were performed using the Pileup algorithm of the Wisconsin PackageTM and figures were prepared with the BOXSHADE 3.21 program. The phylogenetic analysis was done by protein distance as described in PHYLIP v3.695.

### Fish husbandry

Zebrafish embryos were obtained by crossing Cologne (KOLN) strain wild type adult fish, raised at 28°C and staged as described (33). Animal protocols were approved by the veterinary agency of North-Rhine Westphalia (Landesamt für Natur, Umwelt und Verbraucherschutz [LANUV], Germany).

### Expression and purification of recombinant zebrafish Comp and generation of specific antibodies

A cDNA construct encoding mature zebrafish Comp was generated by RT-PCR on whole zebrafish mRNA. Suitable primers introduced 5´ terminal NheI (5’caatgctagccaaggaatatctagagatggagag3’) and 3´ terminal BamHI restriction sites (5’caatgcggccgcttaaaaaagctggatttgctg3’). The amplified PCR product was inserted into a modified pCEP-Pu vector (34) containing an N-terminal BM-40 signal peptide and a N-terminal Twin-Strep-tag upstream of the NheI restriction site. The recombinant plasmid was introduced into human embryonic kidney 293-EBNA cells (Invitrogen) using the FuGENE 6 transfection reagent (Roche). The cells were selected with puromycin (1 µg/mL) and when expressing the Strep-tagged protein transferred to serum free medium for harvest. After centrifugation, the cell culture supernatants were adjusted to pH 7.4, applied to a StrepTactin column (IBA) and eluted following the supplier’s protocol.

The purified recombinant zebrafish Comp was used to immunize a rabbit and a guinea pig. The antisera were purified by affinity chromatography on a column with antigen coupled to CNBr-activated Sepharose (GE Healthcare). The specific antibodies were eluted with 0.1 M glycine, pH 2.5, the eluate neutralized with 1 M Tris-HCl, pH 8.8, and adjusted to 150 mM NaCl. An immunoblot of the recombinant protein detected with the affinity-purified antibodies is shown in **Supplementary Figure 1**.

### Immunoprecipitation

For immunoprecipitation of endogenous Comp, whole 5 dpf zebrafish embryos were homogenized with a Dounce homogenizer and lysed for 30 min on ice in a solution containing 50 mM Tris-HCl, pH 7.4, 150 mM NaCl, 2 mM EDTA, 1% Nonidet P-40, and protease inhibitors. Tissue extracts were centrifuged and the supernatants recovered. Samples (1.5 mL) were first precleared by incubation for 5 h at 4°C with 50 μL of protein A-Sepharose (Roche). They were then centrifuged and the supernatants incubated overnight at 4°C with 4 μg affinity purified polyclonal guinea pig antibodies and 50 μL protein A-Sepharose. After centrifugation at 500 × g the beads were washed once with extraction buffer and twice with TBS, eluted in 2× Laemmli sample buffer containing β-mercaptoethanol, boiled for 3 min and analyzed by SDS-PAGE and western blot. For peptide mass fingerprint analysis gels were stained with Coomassie Brilliant Blue, bands cut out and MALDI-TOF mass spectrometry carried out by the Central Bioanalytic Core Unit in the Center for Molecular Medicine Cologne, University of Cologne, using a standard procedure (35, 36).

### Protein extraction, SDS-PAGE, agarose/polyacrylamide composite gel electrophoresis and immunoblot

For one step protein extraction, zebrafish larvae were homogenized in 10 volumes 50 mM Tris, pH 7.4, 150 mM NaCl, 2 mM EDTA, 1% Nonidet P-40 and protease inhibitors, and put on ice for 15 min. 1/3 volume of 4x SDS Laemmli sample buffer (8% (w/v) SDS, 40% (v/v) glycerol, 0.2% (w/v) bromphenol blue, 250 mM Tris-HCl, pH 6.8) was added, the samples boiled for 5 min, centrifuged for 10 min and subjected to 4-10% (w/v) gradient gel SDS-PAGE. For sequential extraction, 178 wild type zebrafish larvae were frozen at -80°C. On the day of extraction, the larvae were thawed, 1.78 mL of chilled buffer I (0.15 M NaCl, 50 mM Tris-HCl, pH 7.4) (TBS) was added, the fish homogenized with a Dounce homogenizer and extracted on a roller shaker at 4°C for 10h. The extracts were clarified by centrifugation and the supernatants stored at -20°C. The pellets were re-extracted in an identical manner with buffer II (1 M NaCl, 10 mM EDTA, 50 mM Tris, pH 7.4) and the remaining insoluble material was extracted with buffer III (8M urea, 10 mM EDTA, 50 mM Tris, pH 7.4). All extraction buffers contained Complete protease inhibitor (Roche). For agarose/polyacrylamide composite gel electrophoresis samples were supplemented with Laemmli SDS-sample buffer and urea to a final concentration of 2 M and subjected to electrophoresis on 0.5% (w/v) agarose/3% (w/v) polyacrylamide composite gels (37). Proteins were transferred to a nitrocellulose membrane and probed using affinity-purified polyclonal antibodies diluted in TBS/5% milk powder. Bands were detected by chemiluminescence using a peroxidase-conjugated swine anti-rabbit IgG secondary antibody (Dako).

### Generation of zebrafish Comp mutants using CRISPR/Cas9

A guide RNA (gRNA) for *comp* (ENSDART00000171255) targeting the 5’- end of exon 8 (5’-GGAACTGACACCGATATCGATGG-3’, 874-896 nt) was designed using the software CHOPCHOP (https://chopchop.cbu.uib.no/). The synthesis of target oligonucleotides (Eurofins Genomics) and the preparation of gRNAs were carried out as described (38). For the Cas9 mRNA *in vitro* transcription, the pT3TS-nCas9n vector (Addgene) was linearized by XbaI (New England BioLabs) digestion and purified using the Nucleospin Gel and PCR Clean-up Kit (Macherey-Nagel). DNA was transcribed using mMESSAGE mMACHINE T3 Kit (Invitrogen). mRNA polyadenylation was performed using the Poly(A) Tailing Kit (Ambion) and the Cas9 transcript was purified by RNeasy Mini Kit (Qiagen, Germany). The mRNA quality was checked by electrophoresis on a 1% (w/v) formaldehyde agarose gel. The gRNA (12.5 ng/μL) and Cas9 mRNA (300 ng/μL) were mixed in Danieau solution (58 mM NaCl, 0.7 mM KCl, 0.4 mM MgSO_4_, 0.6 mM Ca(NO_3_)_2_, 5 mM Hepes, pH 7.6) with a tracer dye (0.5 mg/mL dextran conjugated with tetramethylrhodamine, Molecular Probes) in a final volume of 5 μL and pre-heated at 60°C for 10 min. Microinjection was carried out using an InjectMan micromanipulator (Eppendorf) assembled on a Leica M165 FC stereomicroscope. After 24 h the DNA from single embryos was extracted by proteinase K digestion (2.5 mg/mL, Sigma Aldrich) in lysis buffer (100 mM Tris HCl, pH 8.5, 5 mM EDTA, 0.2% (w/v) SDS, 200 mM NaCl) overnight (o/n) at 55°C, followed by isopropanol precipitation and resuspension in 20 mM Tris-HCl, 1 mM EDTA, pH 8.0. To evaluate targeting efficiency T7 Endonuclease Assay was performed (28). For genotyping the 283 bp amplicon obtained using 5’-CGCAATGGAAACAAACTGATTA-3’ forward and 5’- GAGTTTGGAACAGTGAGGCAAT-3’ reverse primers was digested with ClaI enzyme.

### qPCR

RNA was extracted from RNA pools of 20 embryos at different stages of development (5, 12 and 24 hours post fertilization, hpf; 2, 3 and 4 days post fertilization, dpf, in duplicate) using Qiazol (Qiagen) and DNase digestion (Invitrogen) according to manufacturer’s instructions. RNA quantity was determined by NanoDrop spectrophotometer and RNA quality by agarose gel electrophoresis. Reverse-transcription was performed using the High-Capacity cDNA Transcription kit (Applied Biosystems) according to manufacturer’s protocol in a final volume of 20 µL. qPCR was performed in triplicate in a 25 µL final volume with SYBR Green Master mix (Applied Biosystems) using the QuantStudio 3 thermocycler and the QuantStudio Design & analysis software (Applied Biosystems).

The following primers were used: for Comp (ENSDART00000171255_2) forward, 5’-GAGTGTGAAGCCTGTGGCAT-3’ and reverse, 5’-CACTCGTCAACGTCCTCACA-3’ (334-353 nt), for β-actin (ENSDART00000054987_7) forward, 5- GAAGATCAAGATCATTGCTCCCCC -3’ and reverse, 5’- GTTCTGTTTAGAAGCACTTCCTGTG -3’ (1110-1135 nt) and for dna15ta1 forward, 5’-TACTGTGCTCAAATTGCTTCA-3’ and reverse, 5’-AATGAGTACTGTGAACTTAATCCAT-3 ‘ (39). The annealing temperature was 60°C for the normalization with β actin and 58°C for the normalization with dna15ta1. ΔΔCt was used for quantitation.

### Tissue labelling procedures

Zebrafish larvae were fixed overnight at 4°C in 4% paraformaldehyde (PFA) in PBS, pH 7.4, washed in PBS containing 0.1% Tween and finally washed and stored in methanol at −20°C. To bleach pigment and block endogenous peroxidases, larvae were incubated overnight in 3 mL of 10% H_2_O_2_ in methanol, then 10 mL of phosphate buffered tween (PBT) was added and the incubation continued for further 16 to 24 h. Larvae were washed in PBT, digested with 2 μg/mL proteinase K for 8 min and fixed again in 4% PFA for 15 min. After washing, larvae were treated with bovine testicular hyaluronidase (Sigma; 500 units/mL in 0.1 M NaH_2_PO_4_, 0.1 M sodium acetate, pH 5.0) at 37°C for 2h and blocked in 3% normal goat serum for 2 h. Affinity purified rabbit polyclonal antibodies were applied at appropriate dilutions and the specimens incubated for 2 h. The primary antibodies were visualized by consecutive treatment of larvae for 2 h each with biotinylated secondary antibody and a streptavidin–peroxidase conjugate (ABC kit, Vectastain). All antibodies were diluted in 3% (w/v) normal goat serum in PBT. For color development, larvae were pre-soaked in diaminobenzidine (0.2 mg/mL PBT) for 30 min and 1 μL of 0.3% H_2_O_2_ solution was added while the larvae were observed under a dissection microscope. For whole-mount immunofluorescence analysis zebrafish were incubated with primary antibody overnight at 4°C, washed four times for 25 min at room temperature, incubated for 2 h at room temperature with fluorescently labelled secondary antibody and washed again four times. The embryos were cleared in 80% glycerol.

Paraffin-embedded sections were deparaffinized. After rehydration in PBS, the sections were digested with bovine testicular hyaluronidase (Sigma, 500 units/mL in 0.1 M NaH_2_PO_4_, 0.1 M sodium acetate, pH 5.0) at 37°C for 30 min and incubated with 0.2% Triton X-100 in TBS for 10 min. After washing, the sections were blocked with 1% (w/v) bovine serum albumin in TBS for 1 h and incubated with the affinity-purified rabbit polyclonal antibody overnight at 4°C. The primary antibodies were visualized by consecutive treatment with biotin-SP-conjugated goat anti-rabbit IgG (Dianova) and alkaline phosphatase-conjugated streptavidin (Dianova) for 1 h each. Antibodies and enzyme-conjugates were diluted in 1% (w/v) bovine serum albumin in TBS and the slides developed with Sigma FAST™ Fast Red TR/naphthol AS-MX (Sigma).

### Electron microscopy

Zebrafish were fixed with 2% glutaraldehyde in 0.1 M sodium cacodylate, pH 7.2-7.3 overnight at 4°C and washed three times with PBS for 10-20 min. Thereafter, tissue was osmicated with 1% OsO4 in 0.1 M cacodylate and dehydrated in increasing ethanol concentrations. Araldite infiltration and flat embedding were performed following standard procedures. Toluidine blue was used to stain semithin sections of 0.5 µm. 70-nm-thick sections were cut with an Ultracut UCT ultramicrotome (Reichert) and stained with 1% aqueous uranylic acetate and lead citrate. Samples were analyzed with a Zeiss EM 109 electron microscope (Zeiss).

## Results

### The zebrafish genome contains duplicated genes coding for Thbs1-4 and a single gene coding for Comp

**Figure 1:**
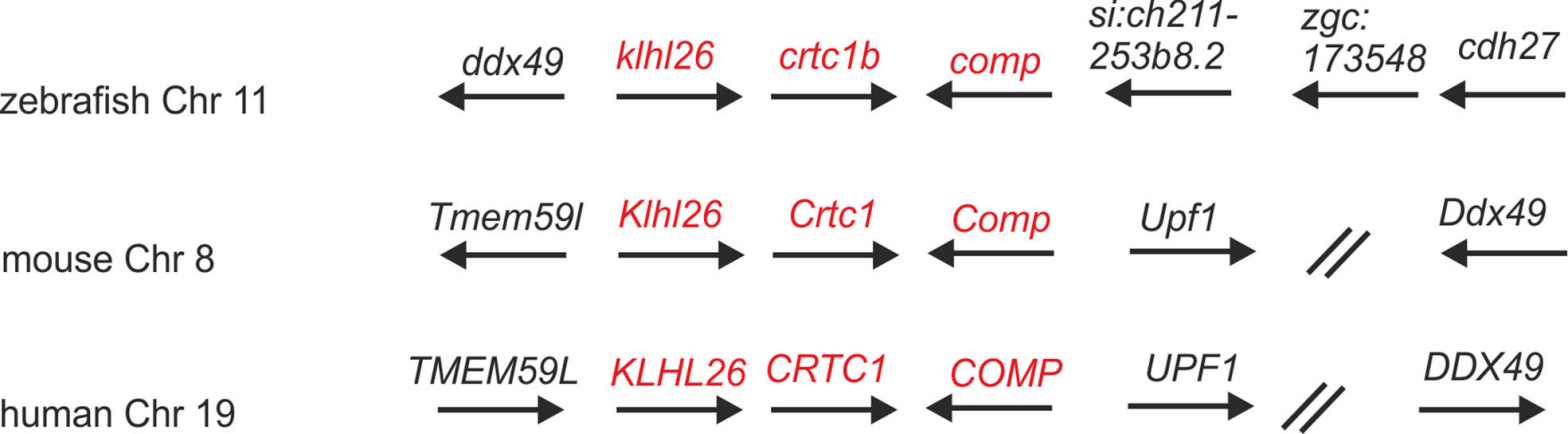
Syntenic relationships of Comp gene loci in zebrafish, mouse and human. Arrows indicate the 5’ to 3’ orientation of the genes. COMP genes and syntenic genes in the direct neighborhood are in red. Note that synteny is only present downstream of the Comp genes. ZFIN gene designations are used for genes lacking a gene name. The double slash indicates regions in the human and mouse genomes (52 and 29 Mbp, respectively) lacking synteny. Note, although not in the same order and direction, the ddx49 genes in zebrafish, mouse and human are all in the vicinity of the COMP genes.

In the most recent version of the zebrafish genome database (GRCz11) and in ZFIN (https://zfin.org/) nine thrombospondin genes are listed, eight duplicated genes coding for thrombospondin 1-4 and a single gene coding Comp (**Supplementary Table 1**). *comp* is located on chromosome 11 (6,941,389-6,974,022 reverse strand) and *crtc1b* and *klhl26* which are located downstream of comp are in synteny with their ortholog genes in human and mouse (**Figure 1**). Like the genes coding for COMP in man and mouse, the zebrafish *comp* gene contains 19 coding exons and the intron-exon structure and the phase of the introns are completely conserved. The length of five exons is slightly different between zebrafish and mouse (**Supplementary Table 2**).

### The protein sequence of zebrafish Comp is highly conserved and most similar to that of zebrafish Tsp4b

**Figure 2:**
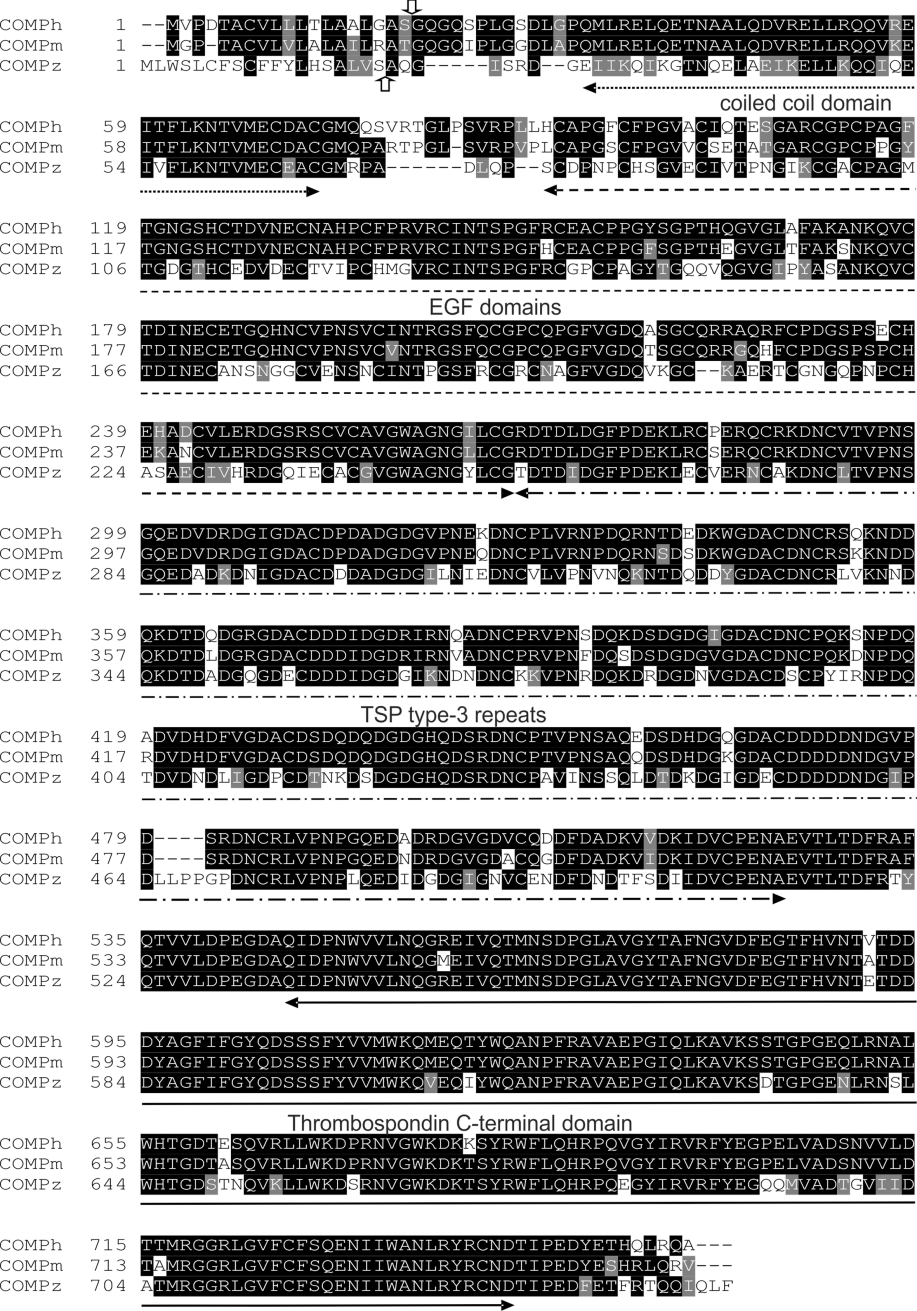
Alignment of amino acid sequences of the human, mouse and zebrafish COMP. The vertical arrows mark the potential signal peptide cleavage sites (up, zebrafish; down, human and mouse). The positions of the domains are indicated by horizontal arrows.

**Figure 3:**
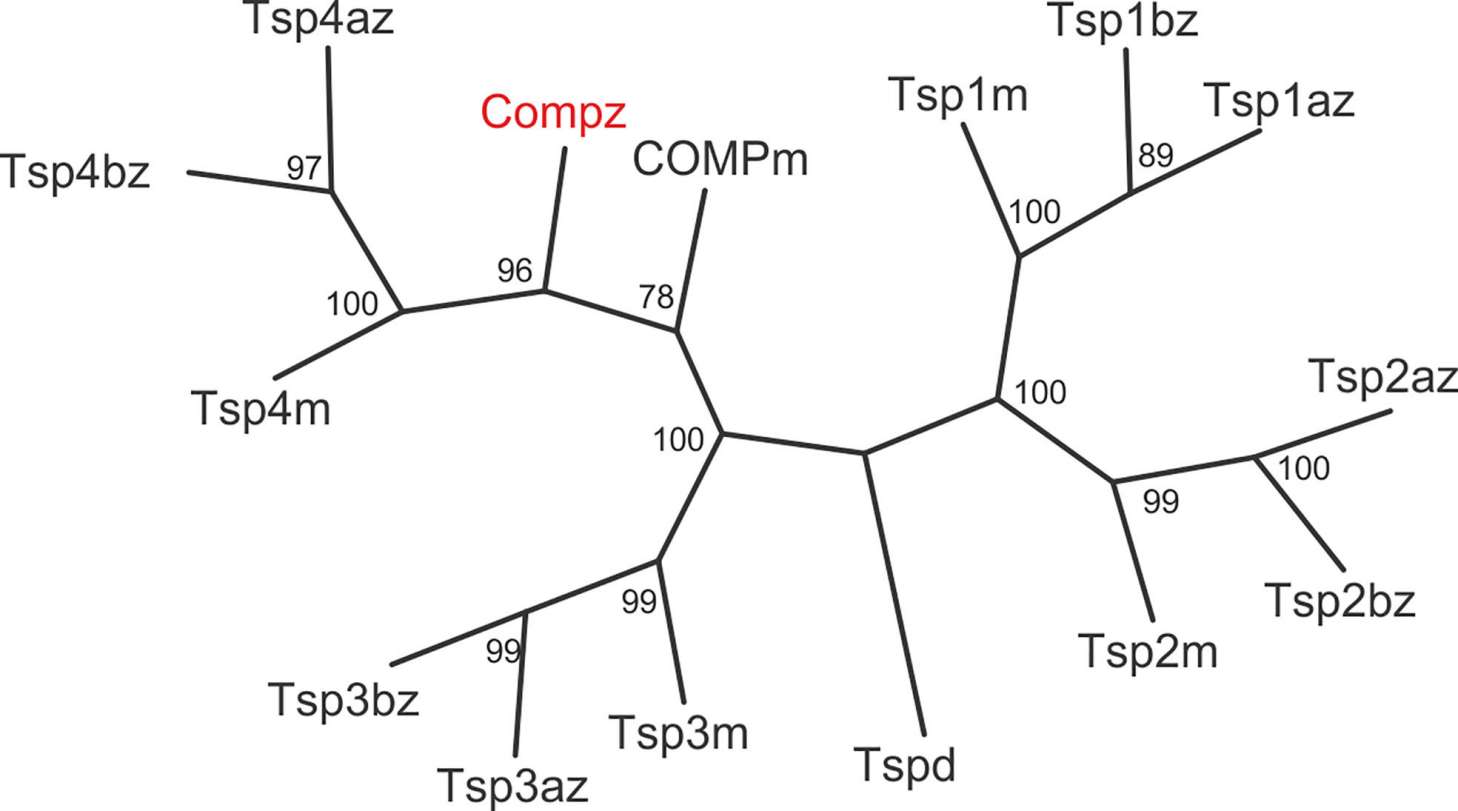
Phylogenetic tree of thrombospondins. Thrombospondin sequences comprising the EGF domains, TSP type-3 repeats and the C-terminal domains from zebrafish *(z)* and mouse *(m)* were aligned using the PILEUP program of the GCG package, using the default parameters. As a full-length sequence for zebrafish thrombospondin 1a was not present in the databases, we amplified the lacking N-terminal sequence by RT-PCR (see **Supplementary Figure 4**). The tree was constructed using the programs PROTEIN DISTANCE, Fitch-Margoliash and CONSENSE of the PHYLIP package version 3.695. Bootstrap analyses using 100 replicates were performed to show the significance. The numbers indicate the statistical weight of the individual branches. Drosophila *(d)* thrombospondin was used as outgroup.

The zebrafish Comp protein consists of 749 amino acid residues (NP_001313279.1), the overall similarity to the murine COMP is 74% and the identity is 67% (**Figure 2**). An isoform lacking a single glycine residue as the consequence of alternative splicing is listed in the database. This glycine residue is located in the linker between the coiled coil and the first EGF like domain. However, the variant is most likely a minor form, as it is found only in a single myoblast EST clone (CT608499.2). The domain structure of zebrafish Comp is completely conserved, consisting of an N-terminal coiled-coil domain, followed by four EGF-like domains, eight TSP type-3 repeats and the C-terminal domain. The predicted signal peptide sequence contains 20 amino acid residues (32) and the mature main isoform has a molecular mass of 79748 Da. All cysteine residues in the mature protein are conserved, while the EGF-like domains are the least, and the C-terminal domain the best conserved. The positions of aspartic acid residues in the TSP type-3 repeats that display a contiguous series of calcium binding sites (40) are, except for one residue, completely conserved. Most similar among the zebrafish thrombospondins is Tsp4b, having 60% similarity and 54% identity to zebrafish Comp. A phylogenetic tree based on the alignment of the protein sequences of the shared parts of the thrombospondins from mouse and zebrafish was constructed by protein distance methods (**Figure 3**). The sequences of the duplicated zebrafish thrombospondins 1-4 are always located in the same branches as the respective mouse thrombospondins, clearly indicating that they are orthologs. Although zebrafish and mouse COMP are not in the Tsp4 sub-branch they are clearly part of the larger TSP4/TSP5 branch.

### Zebrafish Comp forms pentamers and is easily extractable from tissue

**Figure 4:**
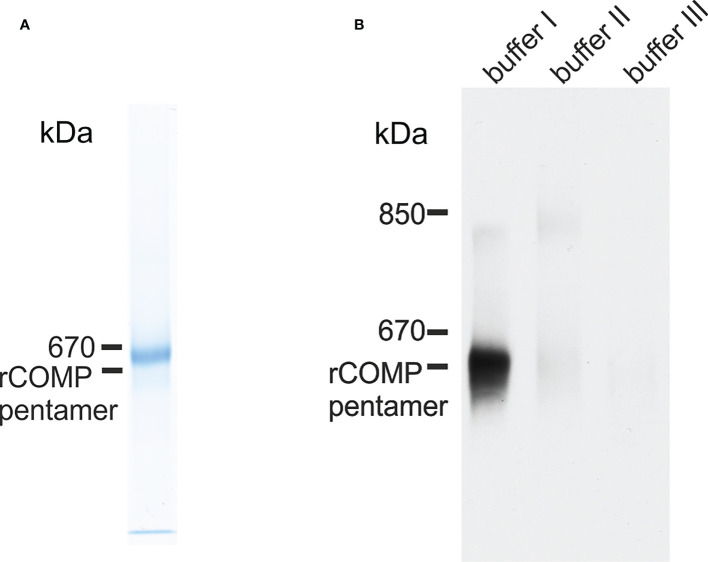
Immunoblot analysis of recombinant zebrafish Comp and Comp in extracts from zebrafish embryos. **(A)** Recombinant zebrafish COMP and **(B)** sequentially extracted proteins from whole 5 dpf zebrafish were separated in 0.5% (w/v) agarose/3% (w/v) polyacrylamide composite gels under non-reducing conditions and detected with guinea pig polyclonal antibodies specific for zebrafish Comp. Buffer I, TBS; buffer II, 50 mM Tris, pH 7.4, 10 mM EDTA, and 1M NaCl, buffer III, 50 mM Tris, pH 7.4, 10 mM EDTA and 8M urea. Recombinant rat COMP was used as a marker and the position of the pentamer is indicated.

Zebrafish Comp was recombinantly expressed in 293 EBNA cells to determine the oligomeric state, as pentameric coiled-coils are not detected by prediction programs, e.g. Multicoil. Indeed, non-reducing composite agarose/SDS-PAGE of the purified recombinant protein showed a prominent band at the calculated mass of a pentamer, similar to human or murine COMP (**Figure 4A**). The purified protein was used to immunize a rabbit and a guinea pig and the antisera purified by affinity chromatography on a column carrying the original antigen. As the quality of the antibodies is crucial, their specificity was tested on the Comp knockout strain sa12473, obtained from the European Zebrafish Resource Center (EZRC), as negative control. Sa12473 fish carry a stop codon in exon 13 of the *comp* gene. When applying the affinity purified antibodies, immunoblot bands were not visible for homozygous mutants in contrast to heterozygous and wild type siblings (**Supplementary Figure 1**). Also by immunofluorescence only heterozygous and wild type, but not homozygous Sa12473 fish were stained (**Supplementary Figure 2**) demonstrating the specificity of the antibodies. As the gels were run under reducing conditions monomeric Comp was detected. To study the structure of tissue-derived Comp and to identify similarities or differences to mammalian COMP, sequential extracts from 5 dpf wild type whole zebrafish were analyzed by immunoblotting using the affinity-purified antibodies. Nearly all Comp was extracted with TBS (**Figure 4B**). Here, composite agarose/polyacrylamide gel were run under non-reducing conditions to resolve the disulfide linked Comp oligomers. Like the recombinant zebrafish Comp (**Figure 4A**), the tissue Comp was present as pentamers. Further extraction with high salt/EDTA followed by 8M urea did not yield any substantial amounts of Comp (**Figure 4B**). To determine if Comp forms hetero-oligomers with Tsp4 (41) also in zebrafish, immunoprecipitation experiments were performed on 5 dpf wild type whole zebrafish TBS extracts using Comp antibodies. Indeed, analysis of the immunoprecipitates by peptide mass fingerprint mass spectrometry revealed that the only thrombospondin that co-immunoprecipitated with Comp (**Supplementary Table 3**) was Tsp4b, as seven specific peptides were detected (**Supplementary Table 4**), but no peptides derived from other thrombospondins (**Supplementary Table 5**).

### Zebrafish Comp is strongly expressed in myosepta and notochord but not in cartilage

**Figure 5:**
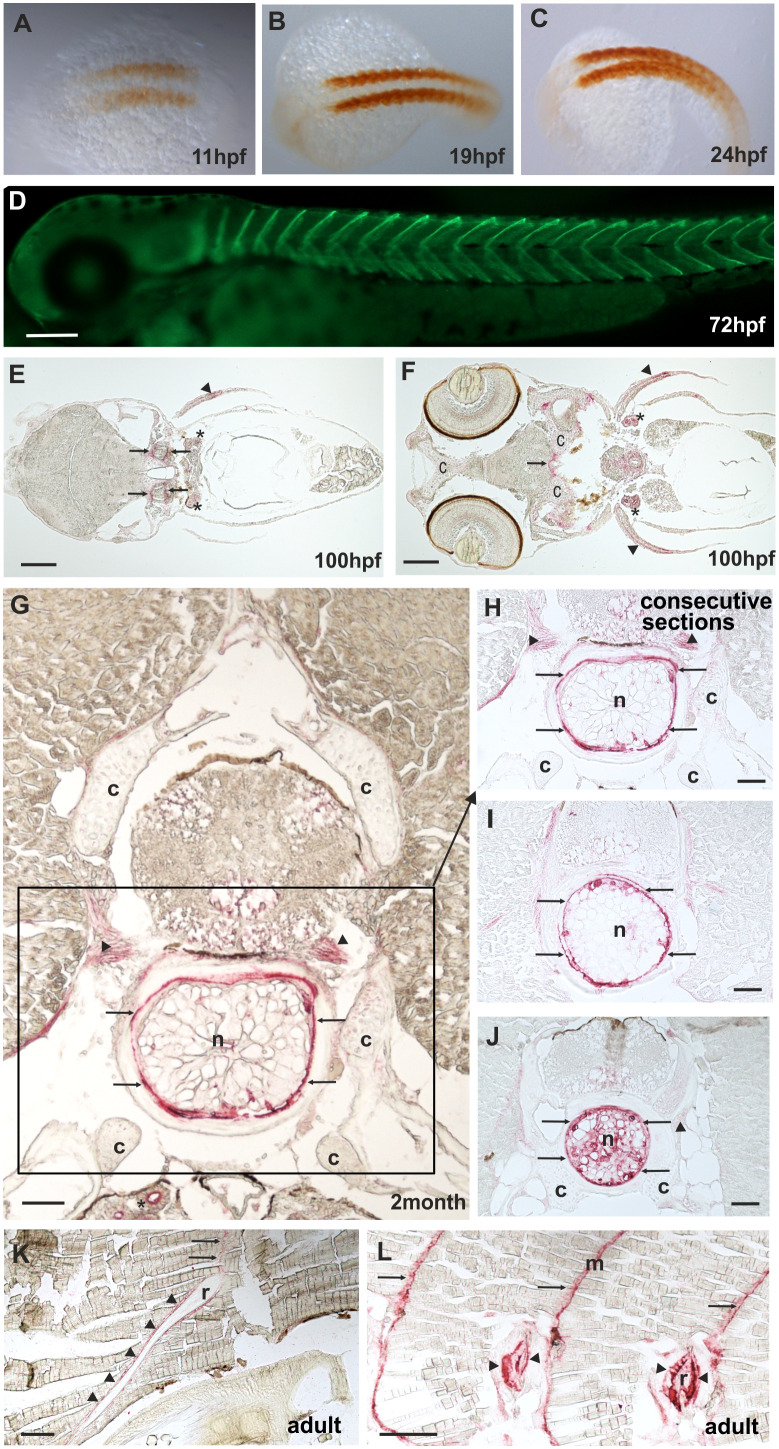
Comp distribution in zebrafish tissues at different stages of development. Immunostainings were performed using an affinity-purified rabbit antiserum directed against zebrafish Comp. **(A–D)**, whole mount staining of 11hpf **(A)**, 19hpf **(B)**, 24 hpf **(C)** and 72 hpf **(D)** zebrafish. At 11-24 hpf Comp is expressed in the somites (**A–C**, brown peroxidase staining) and at 72 hpf in the myosepta (**D**, green fluorescence). **E-L**, immunostaining was carried out on paraffin-embedded tissue sections from 100 hpf **(E, F)**, 2-month-old **(G–J)** and adult **(K, L)** zebrafish by alkaline phosphatase-conjugated streptavidin and Fast Red staining. In the head, at 100 hpf Comp is expressed in tendinous tissues (*arrows*), but not in cartilage (*c*) **(E, F)**. Comp is also found in the pectoral fin (arrowheads). A transverse section through a vertebral body displays Comp in the notochord (framed) most strongly in the notochord sheath (*arrows*), but not in vertebral cartilage (*c*) of 2-month-old zebrafish **(G, H, J)**. Consecutive transverse sections **(H-J)** show a variable expression around vacuolated notochord cells, most prominent at positions with a narrow notochord **(J)**. Comp was also found in tendinous structures that attach muscles to the vertebra **(G, H, J)** (*arrowheads*). In adult zebrafish **(K, L)** Comp is still expressed in myosepta (*m, arrows*) and around ribs (*r, arrowheads*) where the skeletal muscles are attached. (*Asterisks* in **E**, **F**) Unspecific staining of kidney tubules by the secondary antibody, see also **Supplementary Figure 2**. Bars: 50 µm in **(G–J)**, 100 µm in **(E, F, K, L)** and 200 µm in **(D)**.

By RT-PCR, *comp* expression could be detected before 24 hpf (**Supplementary Figure 3**). The spatial expression pattern of Comp protein was studied by whole mount immunostaining and immunohistochemistry on sections, using the same antibodies as for the immunoblots. At 11 hpf Comp is expressed in the somites (**Figures 5A–C**), and at 72 hpf Comp is virtually exclusively and strongly expressed in myosepta (**Figure 5D**). At 100 hpf, Comp was also found in tendinous structures in the head (**Figures 5E, F**). Published *in situ* hybridization data (https://zfin.org/ZDB-GENE-060606-1/expression) confirm the expression pattern at the transcriptional level (42). In zebrafish, Comp was very strongly detected in the notochordal sheath and around vacuolated notochord cells in the intervertebral disc region (**Figures 5G-J**, see also **Figure 7A**). It was most clearly seen in transverse sections at positions where the diameter of the notochord is smaller (**Figure 5J**) Less prominent, Comp was also found in tendinous structures that attach muscles to the vertebra (**Figure 5G-J**), in a narrow zone surrounding ribs (**Figures 5K, L**) and in the myosepta (**Figures 5K, L**). However, in contrast to in mammals, Comp is not expressed in zebrafish articular cartilage or in cartilage anlagen of developing bones (**Figures 5E, G, H**, **J**).

### Generation and characterization of mutant Comp zebrafish lines

**Figure 6:**
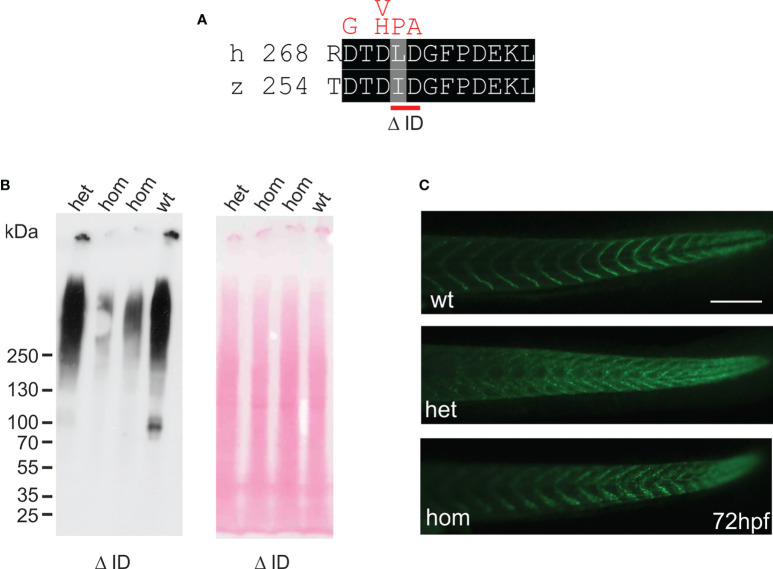
Mutant ΔI258D259Comp is expressed in zebrafish. **(A)** location of the CRISPR-Cas introduced deletion ΔI258D259 (*Δ ID*) in the first TSP type-3 repeat (red bar below) and comparison with mutations in chondrodysplasia patients (46) (above in red). **(B)** immunoblot analysis using an affinity-purified rabbit antiserum specific for zebrafish Comp (left) of direct extracts from 2-month-old wild type (*wt*), and heterozygous (*het*) or homozygous (*hom*) ΔI258D259Comp (*Δ ID*) zebrafish that were submitted to electrophoresis on 4-10% gradient SDS-polyacrylamide gels under non-reducing conditions. Ponceau staining shows equal loading (right). **(C)** whole mount immunofluorescence (green) staining of 72 hpf wild type (*wt*) (n=6), or heterozygous (*het*) (n=10) and homozygous (*hom*) (n=4) ΔI258D259Comp zebrafish mutants were performed using an affinity-purified guinea pig antiserum specific for zebrafish Comp and detected patchy Comp deposition in myosepta of heterozygous and homozygous mutants. Bar: 150 µm.

**Figure 7:**
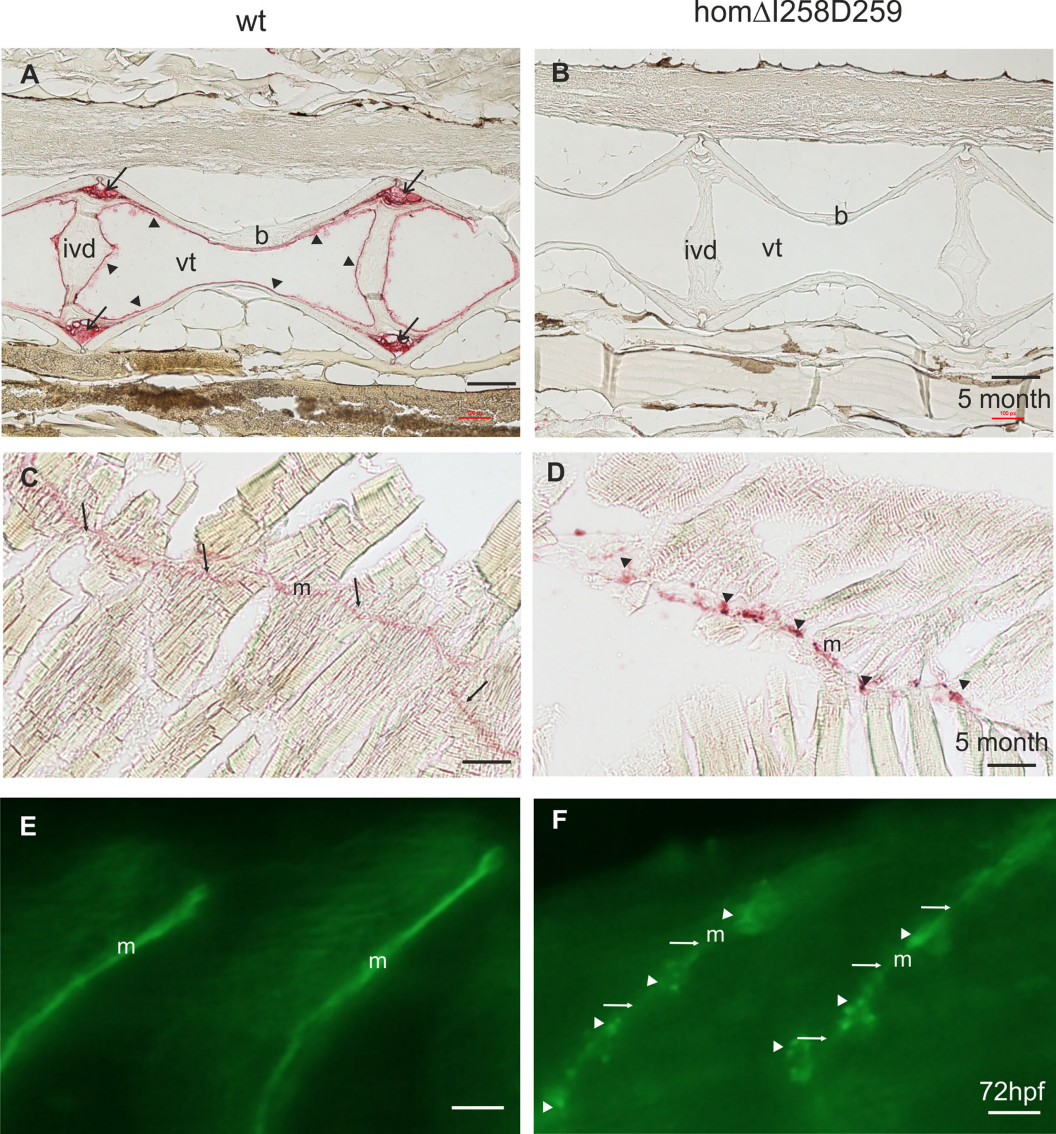
Mutant ΔI258D259Comp expression in vertebral column and muscle of zebrafish. Comp immunostainings were performed on paraffin-embedded tissue sections **(A-D)** from 5-month-old wild type (wt) **(A, C)** (n=4) and homozygous ΔI258D259Comp **(B, D)** (n=4) zebrafish by using affinity-purified rabbit **(A-D)** and guinea pig **(E, F)** antisera specific for zebrafish Comp, alkaline phosphatase-conjugated streptavidin and Fast Red staining **(A-D)** and secondary Alexa 488 conjugated antibodies **(E, F)**. **(A)** In the vertebral column of 5-month-old wt zebrafish, Comp is found in the fibrocartilaginous tissue at the base of the intervertebral discs (*ivd*) (*arrows*) and lining the inner part of the bones (b) (*arrowheads*), (*vt*, vacuolated tissue). **(B)** In mutant ΔI258D259Comp zebrafish this expression is nearly completely lacking. However, the architecture of the vertebral column is not altered. **(C)** In adult wild type zebrafish Comp is still uniformly found in myosepta (m). **(D)** Also in adult mutant ΔI258D259Comp zebrafish Comp is found in myosepta (*m*) but with an irregular patchy distribution (*arrowheads*). **(E, F)** This is similar in 72 hpf zebrafish where Comp is strongly and uniformly found in myosepta (*m*) of wt **(E)** and in irregular patches (*arrowheads*) interrupted by a much weaker uniform staining (*arrows*) in ΔI258D259Comp zebrafish as shown by whole mount immunofluorescence staining using an affinity-purified guinea pig antiserum specific for zebrafish Comp **(E, F)** (n=100), see also **Figure 6C**. Bars: 100 µm in **(A-D)** and 10 µm in **(E, F)**.

**Figure 8:**
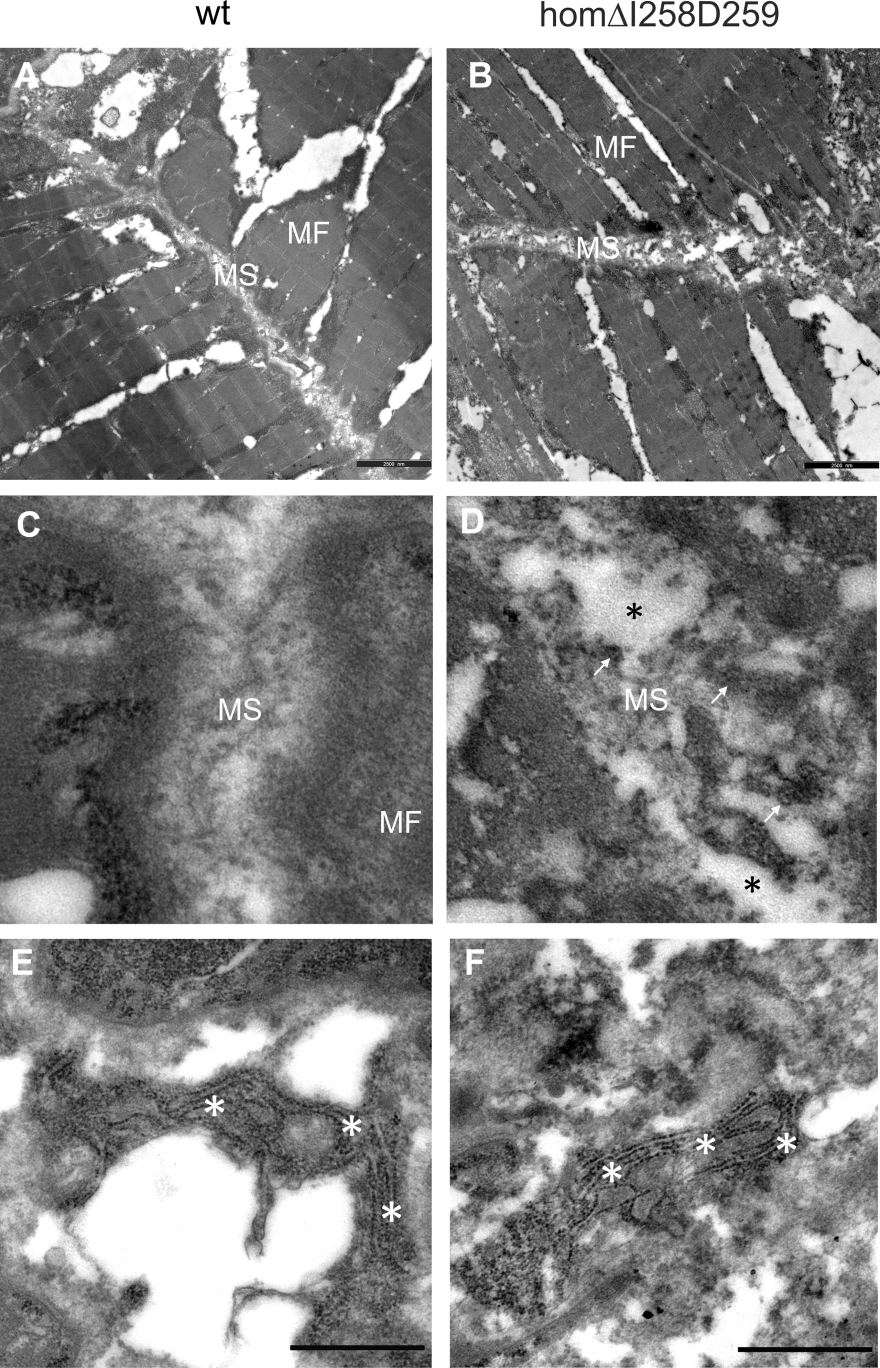
Electron microscopy of myosepta in wild type and mutant ΔI258D259Comp skeletal muscle of 72hpf zebrafish. The skeletal muscle fiber (*MF*) structure is comparable in wild type (*wt*) **(A)** and mutant ΔI258D259Comp **(B)** zebrafish while the myosepta (*MS*) in mutants are less dense and slightly enlarged compared to control. At higher magnification the myosepta of wild type **(C)** show a largely homogenous extracellular matrix (ECM) structure while myosepta of mutants **(D)** show disrupted (*black asterisks*), inhomogeneous and clustered ECM (*arrows*) structure with different density. The fibroblasts in wild type **(E)** and mutants **(F)** show an extensive endoplasmic reticulum (*white asterisks*) without obvious differences, indicating intact synthesis- active fibroblasts and no significant retention of mutant protein.

In humans, mutations in the *COMP* gene lead to pseudoachondroplasia (PSACH) and multiple epiphyseal dysplasia (MED) (43). Mutations result either in retention of COMP in the rough endoplasmic reticulum (rER) of chondrocytes and subsequently premature chondrocyte death (44) or in the secretion of a dysfunctional COMP disturbing ECM assembly (23, 45). Although zebrafish do not express Comp in cartilage, due to the broad expression in larval myosepta they could represent a model both to study the consequences of COMP mutations and to test therapeutic approaches. Indeed, large scale screening is possible as the expression in myosepta can be easily monitored by whole mount immunofluorescence. A sequence encoding a calcium binding site in the first TSP type-3 repeat was targeted by CRISPR-Cas9 gene editing, to generate zebrafish Comp mutants. This TSP type-3 repeat was chosen, as mutations of the equivalent amino acid residues L272 and D273 (**Figure 6A**) are linked to PSACH and the D273 is proposed to be involved in Ca^2+^ binding (46). Four lines with frameshift mutations and two lines with small in frame deletions were generated. The lines with frameshift deletions were, as the sa12473 line (**Supplementary Figure 1**), complete Comp knockouts (**Supplementary Figure 1**). A line (ΔI258D259Comp) with an in-frame deletion resulting in the lack of I258 and D259 was further characterized. The homozygous and heterozygous mutant fish are viable and do not show an obvious phenotype (not shown). Immunoblotting of extracts from 2-month-old zebrafish revealed a slightly reduced Comp expression in heterozygous and a stronger reduction in homozygous mutant zebrafish, indicating that mutant ΔI258D259Comp is still expressed (**Figure 6B**). Interestingly, a band at approx. 100 kDa, most likely representing Comp lacking its coiled coil oligomerization domain, is lacking in heterozygous and homozygous fish (**Figure 6B**). Although Comp is also expressed in the notochord, the general architecture of the vertebral column in homozygous adult zebrafish was unaffected (**Figure 7B**), Interestingly, in wild type zebrafish Comp is strongly present in the fibrocartilaginous tissue in a very narrow zone at the base of the intervertebral discs and also lining the bone‐shaped vacuolated tissue (**Figure 7A**). Whole mount immunofluorescence staining on 72 hpf zebrafish larvae from heterozygous breedings showed only two phenotypes. In one, the Comp antibody strongly and continuously stains the myosepta and irregularities are hardly seen. In the other phenotype, the myosepta are also stained, but the continuous staining is much weaker and, in contrast, irregular spots of strong staining appear within the myosepta indicating an irregular deposition of mutant Comp (**Figure 6C**). This pointed to a dominant phenotype and indeed genotyping revealed that only heterozygous and homozygous mutant zebrafish showed the distinct, patchy staining pattern of Comp (**Figure 6C**). Comp is absent in homozygous fish, but, as Comp forms pentamers, also in heterozygous mutant animals the presence/amount of pure wild type COMP is neglectable. Further, the similar staining pattern indicates that mutant ΔI258D259Comp pentamers and hybrid pentamers consisting of wild type and mutant ΔI258D259 Comp behave in a similar manner. Like in 72 hpf (**Figures 7E, F**), also in 5-month-old zebrafish mutant ΔI258D259 Comp is not continuously deposited within the myosepta, as is wild type Comp, and shows a patchy distribution (**Figures 7C, D**). To study the mutant phenotype at higher resolution, electron microscopy was performed on myosepta and adjacent muscle areas in 72 hpf homozygous mutants (**Figure 8**). The organization and structure of muscles adjacent to myosepta were not altered (**Figures 8A, B**) while the myosepta in the mutant fish appear more irregular and slightly enlarged compared to control. At higher magnification (**Figures 8C, D**), the disorganized ECM of the myosepta of mutant fish became obvious. In contrast to the largely homogenous myoseptal ECM of wild type, the myosepta of mutant fish revealed a disrupted, inhomogeneous and clustered ECM with different density. This may explain the patchy staining pattern while the structure of the endoplasmic reticulum of myoseptal synthesis-active fibroblasts appeared unaltered (**Figures 8E, F**).

## Discussion

COMP, a member of the thrombospondin family of ECM proteins, was first isolated from articular and tracheal cartilage as well as from the Swarm rat chondrosarcoma and was considered to be an abundant extracellular structural protein in cartilage (1–3). However, in recent years a much broader tissue distribution became obvious, including expression in tendon (4), skin (5), heart and skeletal muscle and vascular smooth muscle (6). It also became evident that COMP plays a role in collagen secretion (10). COMP is found in all vertebrates, but has not been well characterized in the zebrafish, a widely used model organism. Here we revisited more recent zebrafish genome assemblies and databases and unequivocally identified zebrafish Comp as the single ortholog of mammalian COMP. In contrast, ohnologs, duplicated genes that are common in zebrafish due to the whole genome duplication (WGD) that occurred in the last common ancestor in the teleost lineage (47), are found for the rest of the thrombospondin family.

Our characterization revealed that zebrafish Comp is highly conserved at the protein level and like mammalian COMP forms pentamers. However, it was very surprising that, although zebrafish Comp was found in the fibrocartilaginous matrix of intervertebral discs, Comp was not found in articular cartilage or in cartilage anlagen of developing bones. This is in clear contrast to other cartilage matrix proteins, like matrilin-3 (48). These, just as COMP, are linked to chondrodysplasias in humans (43). Our findings indicate that COMP has acquired a novel unique function in the cartilage of tetrapods, perhaps as the higher mechanical load to which land-living animals are exposed to demands a more strongly interconnected ECM. Further, the strong expression in myosepta, a tendon equivalent in zebrafish, may point to the more primordial and common function of COMP. Interestingly, Tsp4b, the closest Comp neighbour in the thrombospondin protein family of zebrafish, has a tissue distribution that is very similar to that of Comp (31).

In fact, our phylogenetic analysis revealed that Comp is found in the same branch as Tsp4 which makes it very likely that COMP originates from a duplication of an ancestral Tsp4 gene before the additional whole genome duplication occurring in teleosts, as proposed by others (30). It was shown that Tsp4 is essential for muscle attachment and ECM assembly at myotendinous junctions in zebrafish (31). In mammals, hetero-oligomers formed between COMP and TSP4 have been found in equine tendons (41), also reflecting a close functional relationship between COMP and Tsp4. Indeed, in the present study we co-immunoprecipitated Tsp4b using the Comp antibodies, indicating that such hetero-oligomers may exist also in zebrafish. Altogether our results indicate that Comp may play a similar role in zebrafish as in other vertebrates, except for its novel role in tetrapod cartilage.

Interestingly, a single thrombospondin is present already in Drosophila and its structure is most related to vertebrate COMP. The large isoform of Drosophila thrombospondin has been shown to form pentamers which indicates an early evolutionary origin of pentameric thrombospondins (49). Its major sites of expression in the Drosophila embryo are the muscle attachment sites and the precursors of the longitudinal visceral muscles. In larval stages it is expressed in wing imaginal discs (50). It was shown that Drosophila thrombospondin is a key ECM protein required for muscle-specific integrin-mediated adhesion to tendon cells. In thrombospondin mutant embryos muscles fail to attach to tendon cells and often aggregate and form ectopic integrin-mediated junctions with neighboring muscles. This leads to a nonfunctional somatic musculature and embryonic lethality (50).

In vertebrates, the absence of single members of the thrombospondin family does not result in severe phenotypes (30). Especially, mice lacking COMP have no obvious phenotype (17), perhaps due to redundancy between thrombospondins (51) or with other ECM proteins like matrilins (52). The zebrafish Comp knockout strains sa12473 (EZRC) and the CRISPR frame shift knockout generated here also have no obvious phenotype (not shown). In contrast, almost all mutations in COMP that lead to chondrodysplasias in humans are dominant missense or in frame deletion/insertion mutations with a broad spectrum of intracellular and extracellular phenotypes ranging from ER retention to collagen fibril organization (23, 53). Mouse strains carrying patient-derived mutations have been established and were helpful in the characterization of chondrodysplasia pathomechanisms (18, 21). However, use of the mouse system to study pathogenesis and potential treatment can be laborious and expensive. Cell culture models (23, 45) and micro-mass cultures (54) have also been employed, but are physiologically less relevant. The zebrafish, the simplest vertebrate model organism that is also suitable for CRISPR-Cas gene editing, could therefore serve as a valuable alternative to screen for pharmaceutical interventions in analogous PSACH/MED zebrafish Comp mutants. The advantage of this system would be the possibility to screen many compounds/treatments in large numbers of zebrafish larvae on microtiter plates by immunofluorescence microscopy (55). Indeed, in a zebrafish osteogenesis imperfecta model the chemical chaperone 4- phenylbutyrate was shown to ameliorate the skeletal phenotype (56). Recently, the treatment of cystic fibrosis was improved by the development of modulator therapies that target specific cystic fibrosis transmembrane conductance regulator protein malformations (57). Zebrafish has been used to identify altered protein expression by immunohistochemistry in a small molecule screen (58). Here we demonstrate that mutant Comp zebrafish lines will offer the possibility to perform large scale screens for chemical chaperones suitable for correcting misfolded Comp, as we could clearly show that mutant fish can be distinguished from wild type and, therefore, efficacy of treatment can be easily monitored by analyzing the patchy myosepta staining by immunofluorescence microscopy.

Taken together we showed that, although well conserved, Comp is unexpectedly not a cartilage protein in zebrafish. Nevertheless, the expression in tendinous tissue is shared by zebrafish and mammals and the mutant zebrafish phenotype might reflect the tendino- and myopathy described in mice and patients. The easy detection and genomic manipulation by CRISPR-Cas and the very straightforward screening makes the zebrafish a promising model system to study pathogenesis and treatment of COMP-associated tissue pathologies in chondrodysplasias.

## Data availability statement

The datasets presented in this study can be found in online repositories. The names of the repository/repositories and accession number(s) can be found in the article/**Supplementary Material**.

## Ethics statement

The animal study was reviewed and approved by Veterinary agency of North-Rhine Westphalia (Landesamt für Natur, Umwelt und Verbraucherschutz [LANUV], Germany.

## Author contributions

Conceptualization: AF, RW; Data Curation: AF, RW; Funding Acquisition: AF, FZ, MP, RW; Investigation: HF-G, RG, FT, BK, PK, WB; Supervision: AF, MP, RW; Validation: AF, RW; Visualization: WB, RW; Writing - Original Draft Preparation: AF, FZ, RW; Writing - Review and Editing: PK, FT, AF, FZ, MP, RW. All authors contributed to the article and approved the submitted version.

## Funding

This study was supported by the European Community, FP7, ‘Sybil’ project grant No. 602300 and the German Research Foundation grants FOR2722-407164210 (MP and RW).

## Conflict of interest

The authors declare that the research was conducted in the absence of any commercial or financial relationships that could be construed as a potential conflict of interest.

## Publisher’s note

All claims expressed in this article are solely those of the authors and do not necessarily represent those of their affiliated organizations, or those of the publisher, the editors and the reviewers. Any product that may be evaluated in this article, or claim that may be made by its manufacturer, is not guaranteed or endorsed by the publisher.
