# Supplementary figures and images for: Structure, evolution and expression of zebrafish cartilage oligomeric matrix protein (COMP, TSP5). CRISPR-Cas mutants show a dominant phenotype in myosepta
Source: Front Endocrinol (Lausanne). 2022 Nov 14;13:1000662. doi: 10.3389/fendo.2022.1000662 (PMC9702538; doi:10.3389/fendo.2022.1000662)

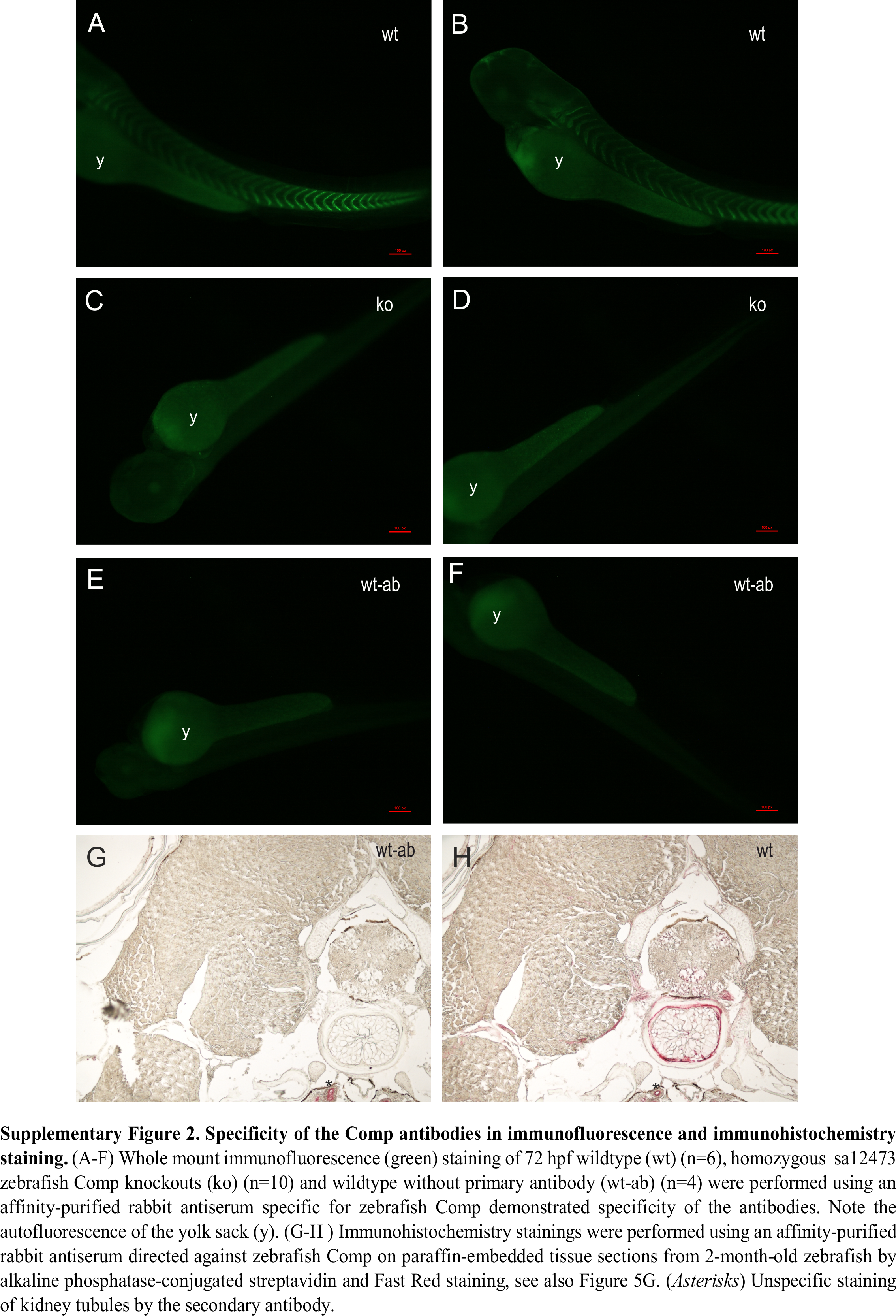

Supplement: Supplementary file 2 [file Image_2.tif]

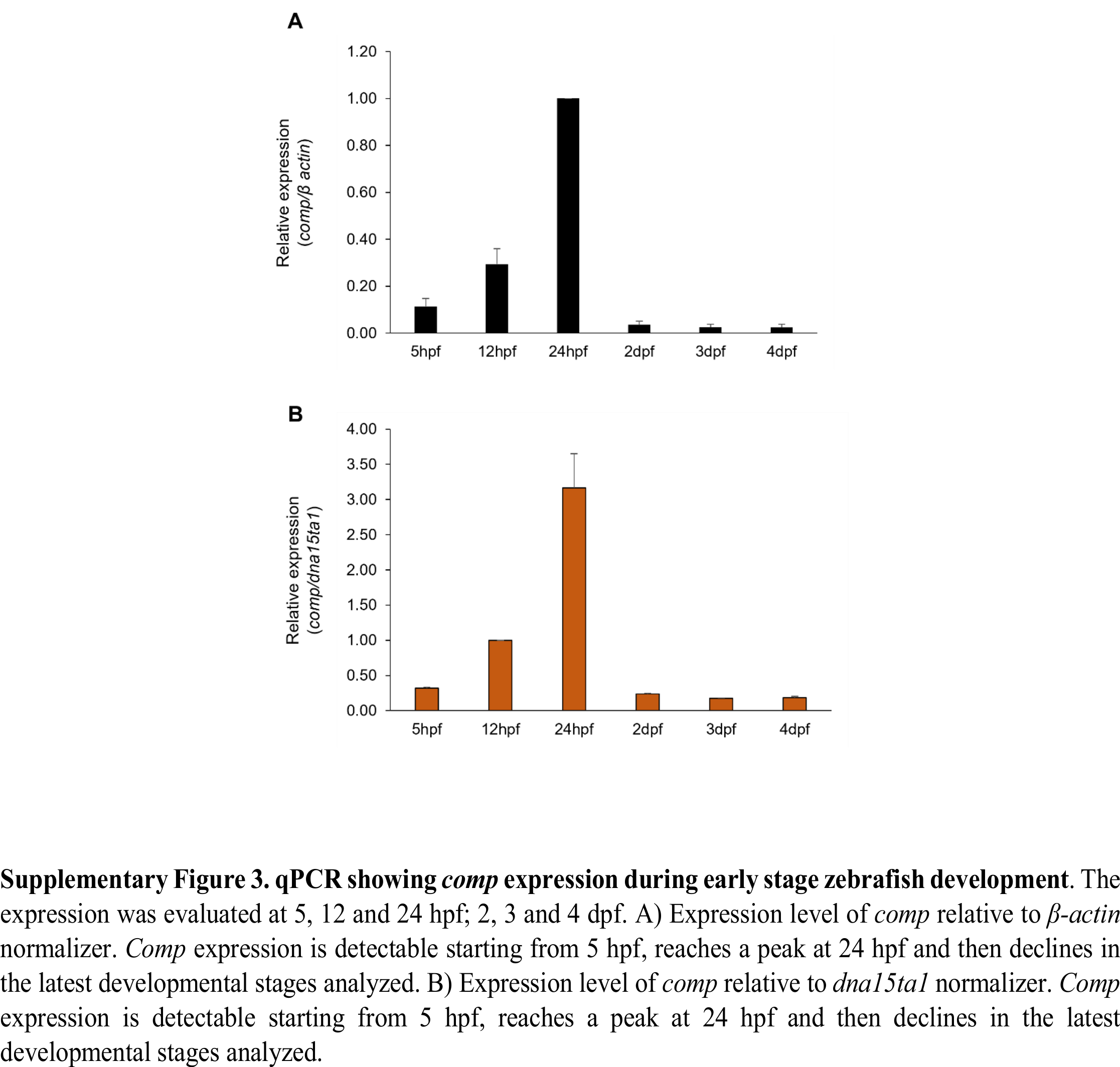

Supplement: Supplementary file 3 [file Image_3.tif]
