# Supplementary material for: Structure, evolution and expression of zebrafish cartilage oligomeric matrix protein (COMP, TSP5). CRISPR-Cas mutants show a dominant phenotype in myosepta
Source: Front Endocrinol (Lausanne). 2022 Nov 14;13:1000662. doi: 10.3389/fendo.2022.1000662 (PMC9702538; doi:10.3389/fendo.2022.1000662)
Supplement: Supplementary file 5 [file Table_1.docx]

**Supplementary Table 1**

Zebrafish thrombospondin genes

| Gene | Chromosome | ENSEMBL | ZFIN |
| --- | --- | --- | --- |
| *thbs1a* | ? (17)* | ENSDARG00000103775 | ZDB-GENE-120402-2 |
| *thbs1b* | 20 | ENSDARG00000010785 | ZDB-GENE-020708-1 |
| *thbs2a* | 13 | ENSDARG00000060410 | ZDB-GENE-020708-2 |
| *thbs2b* | 12 | ENSDARG00000073810 | ZDB-GENE-100623-1 |
| *thbs3a* | 16 | ENSDARG00000077641 | ZDB-GENE-020708-3 |
| *thbs3b* | 19 | ENSDARG00000012060 | ZDB-GENE-060503-84 |
| *thbs4a* | 5 | ENSDARG00000102777 | ZDB-GENE-080215-11 |
| *thbs4b* | 21 | ENSDARG00000020072 | ZDB-GENE-020708-4 |
| *comp* | 11 | ENSDARG00000098431 | ZDB-GENE-060606-1 |

* In the most recent zebrafish reference genome assembly, GRCz11, *thbs1a* is unmapped, in the older Zv9 assembly it was mapped to chromosome 17
