# Supplementary material for: Structure, evolution and expression of zebrafish cartilage oligomeric matrix protein (COMP, TSP5). CRISPR-Cas mutants show a dominant phenotype in myosepta
Source: Front Endocrinol (Lausanne). 2022 Nov 14;13:1000662. doi: 10.3389/fendo.2022.1000662 (PMC9702538; doi:10.3389/fendo.2022.1000662)
Supplement: Supplementary file 6 [file Table_2.docx]

**Supplementary Table 2**

Exon/Intron structure of the COMP encoding genes in zebrafish, mouse and human

|  |  | zebrafish | | | mouse | | | human | | |
| --- | --- | --- | --- | --- | --- | --- | --- | --- | --- | --- |
| Domain | exon | phases | | length | phases | | length | phases | | length |
| Signal peptide | 1 | - | 1 | 76 | - | 1 | 76 | - | 1 | 79 |
|  |  |  |  | 5,149 |  |  | 205 |  |  | 255 |
| Coiled coil | 2 | 1 | 0 | 74 | 1 | 0 | 86 | 1 | 0 | 86 |
|  |  |  |  | 243 |  |  | 434 |  |  | 236 |
| Coiled coil | 3 | 0 | 1 | 52 | 0 | 1 | 52 | 0 | 1 | 52 |
|  |  |  |  | 93 |  |  | 380 |  |  | 447 |
| EGF1 | 4 | 1 | 0 | 149 | 1 | 0 | 170 | 1 | 0 | 173 |
|  |  |  |  | 219 |  |  | 755 |  |  | 644 |
| EGF2 | 5 | 0 | 0 | 138 | 0 | 0 | 138 | 0 | 0 | 138 |
|  |  |  |  | 87 |  |  | 174 |  |  | 246 |
| EGF3 | 6 | 0 | 0 | 75 | 0 | 0 | 75 | 0 | 0 | 75 |
|  |  |  |  | 1,674 |  |  | 80 |  |  | 88 |
| EGF3/4 | 7 | 0 | 0 | 153 | 0 | 0 | 159 | 0 | 0 | 159 |
|  |  |  |  | 81 |  |  | 82 |  |  | 77 |
| EGF4 | 8 | 0 | 0 | 105 | 0 | 0 | 105 | 0 | 0 | 105 |
|  |  |  |  | 97 |  |  | 93 |  |  | 90 |
| EGF4/TSP3 | 9 | 0 | 0 | 108 | 0 | 0 | 108 | 0 | 0 | 108 |
|  |  |  |  | 1,647 |  |  | 353 |  |  | 561 |
| TSP3 | 10 | 0 | 1 | 160 | 0 | 1 | 160 | 0 | 1 | 160 |
|  |  |  |  | 1,135 |  |  | 1,357 |  |  | 840 |
| TSP3 | 11 | 1 | 0 | 119 | 1 | 0 | 119 | 1 | 0 | 119 |
|  |  |  |  | 2,229 |  |  | 165 |  |  | 240 |
| TSP3 | 12 | 0 | 2 | 53 | 0 | 2 | 53 | 0 | 2 | 53 |
|  |  |  |  | 86 |  |  | 83 |  |  | 92 |
| TSP3 | 13 | 2 | 1 | 194 | 2 | 1 | 182 | 2 | 1 | 182 |
|  |  |  |  | 2,179 |  |  | 178 |  |  | 113 |
| TSP3/C-term | 14 | 1 | 0 | 179 | 1 | 0 | 179 | 1 | 0 | 179 |
|  |  |  |  | 2,364 |  |  | 108 |  |  | 126 |
| C-term | 15 | 0 | 1 | 49 | 0 | 1 | 49 | 0 | 1 | 49 |
|  |  |  |  | 82 |  |  | 375 |  |  | 405 |
| C-term | 16 | 1 | 0 | 197 | 1 | 0 | 197 | 1 | 0 | 197 |
|  |  |  |  | 2,583 |  |  | 943 |  |  | 532 |
| C-term | 17 | 0 | 2 | 173 | 0 | 2 | 173 | 0 | 2 | 173 |
|  |  |  |  | 531 |  |  | 254 |  |  | 997 |
| C-term | 18 | 2 | 1 | 140 | 2 | 1 | 140 | 2 | 1 | 140 |
|  |  |  |  | 2,191 |  |  | 74 |  |  | 92 |
| C-term | 19 | 1 | - | 56 | 1 | - | 47 | 1 | - | 47 |

Full conservation, highlighted in yellow; deviation, highlighted in green.

First and last exons, in red, translated sequence incl. stop codon
