# Supplementary material for: Structure, evolution and expression of zebrafish cartilage oligomeric matrix protein (COMP, TSP5). CRISPR-Cas mutants show a dominant phenotype in myosepta
Source: Front Endocrinol (Lausanne). 2022 Nov 14;13:1000662. doi: 10.3389/fendo.2022.1000662 (PMC9702538; doi:10.3389/fendo.2022.1000662)
Supplement: Supplementary file 7 [file Table_3.docx]

**Supplementary Table 3**. Comp peptide fragments detected by ms/ms mass spectrometry analysis after immunoprecipitation with a zebrafish Comp antibody

| 138 | CGPCPAGYTGQQVQGVGIPYASANK | 162 |
| --- | --- | --- |
| 129 | CINTSPGFR | 137 |
| 196 | CNAGFVGDQVK | 206 |
| 382 | DRDGDNVGDACDSCPYIR | 399 |
| 688 | FYEGQQMVADTGVIIDATMR | 707 |
| 641 | NSLWHTGDSTNQVK | 654 |
| 49 | QQIQEIVFLK | 58 |
| 605 | QVEQIYWQANPFR | 617 |
| 522 | TYQTVVLDPEGDAQIDPNWVVLNQGR | 547 |
| 673 | WFLQHRPQEGYIR | 685 |
| 618 | AVAEPGIQLK | 627 |

*Numbering according to NCBI Reference Sequence: NP_001313279.1
