# Supplementary material for: Structure, evolution and expression of zebrafish cartilage oligomeric matrix protein (COMP, TSP5). CRISPR-Cas mutants show a dominant phenotype in myosepta
Source: Front Endocrinol (Lausanne). 2022 Nov 14;13:1000662. doi: 10.3389/fendo.2022.1000662 (PMC9702538; doi:10.3389/fendo.2022.1000662)
Supplement: Supplementary file 8 [file Table_4.docx]

**Supplementary Table 4.** Tsp4b peptide fragments detected by ms/ms mass spectrometry analysis after immunoprecipitation with a zebrafish Comp antibody

| Start^*^ | Sequence | End |
| --- | --- | --- |
| 60 | IQPGTGNTIFSLYNPR | 75 |
| 76 | DNSKYFEFSVFGK | 88 |
| 80 | YFEFSVFGK | 88 |
| 103 | MSAVTFNK | 110 |
| 256 | NTISECQACGLSGAEVVKPK | 275 |
| 336 | CEACPLGFTGKPLEGVGVAYAQTHK | 360 |
| 397 | TGFTGDQIR | 405 |

*Numbering according to NCBI Reference Sequence: NP_775333
